# Upregulation of microRNA-125b by G-CSF promotes metastasis in colorectal cancer

**DOI:** 10.18632/oncotarget.16892

**Published:** 2017-04-06

**Authors:** Xinghua Zhang, Xiao Ma, Huaying An, Changqing Xu, Wenjo Cao, Wei Yuan, Jie Ma

**Affiliations:** ^1^ State Key Laboratory of Molecular Oncology, National Cancer Center/Cancer Hospital, Chinese Academy of Medical Sciences, Peking Union Medical College, Beijing, China; ^2^ Department of Gastroenterology, Shandong Provincial Qianfoshan Hospital, Shandong University, Jinan, China; ^3^ Department of Science, University of British Columbia, Vancouver, Canada; ^4^ Clinical Immunology Center, Chinese Academy of Medical Science, Beijing, China; ^5^ Department of Biotherapy, Beijing Hospital, National Center of Gerontology, Beijing, China

**Keywords:** colorectal cancer, microRNA-125b, MCL1, G-CSF, metastasis

## Abstract

Although there are reports of miR-125b being dysregulated in colorectal cancer (CRC) and associated with CRC progression, little is known about its intrinsic regulatory mechanisms. Here we detected the expression of miR-125b in CRC tissues, subsequently investigated the effect of miR-125b on the proliferation, apoptosis, cell cycle and metastasis on CRC cells. Our results showed that the expression of miR-125b was significantly decreased in CRC tissues comparing to adjacent tissues. However, with the stimulation of Granulocyte colony-stimulating factor (G-CSF), which was highly expressed in CRC tissues, the expression of miR-125b could be improved. Analysis of patient samples revealed that miR-125b presented a clear association with poor differentiation, positive lymph node metastasis, and advanced TNM stage. Overexpression of miR-125b inhibited cell proliferation, triggered G2/M cell cycle arrest, induced subsequent apoptosis, and promoted cell migration and invasion. Moreover, luciferase reporter assays and western blot clarified that the myeloid cell leukemia 1 (MCL1) was a direct target of miR-125b. Thus overexpression of MCL1 attenuated the pro-metastasis function of miR-125b in CRC cell lines. In addition, the protein expression level of MCL1 was decreased in CRC tissues from patients with positive lymph node metastasis, which had high miR-125b expression. Collectively, our study suggested that miR-125b induced by G-CSF plays a promoting role in the metastasis of CRC by targeting MCL1, which may serve as a novel therapeutic target for CRC metastasis.

## INTRODUCTION

Colorectal cancer (CRC) has become the third leading cause of cancer-related death worldwide [1]. Despite advanced treatment modalities in recent decades, the 5-year survival rate of CRC remains unsatisfied [2] because approximately half of CRC patients ultimately develop metastases disease, especially liver metastases [3]. Therefore, a better understanding of molecular mechanisms involved in the progression of CRC is crucial to explore novel therapeutic targets for CRC treatment.

MiRNAs can function as tumor suppressors or oncogenes according to their targets [4]. Although numerous miRNAs have been identified as important in CRC development, there is a limited application to clinical diagnosis and treatment [5]. Therefore, endless efforts are focused on the search of useful miRNAs as diagnosis markers and therapy targets for CRC patients.

MiR-125b is constituted by two homologs (miR-125b-1 and miR-125b-2) whose genes are located in chr11q24 and chr21q21 respectively (http://microrna.sanger.ac.uk/). MiR-125b is thought to act as either oncogene or tumor suppressor which displays a heterogeneitic expression in different carcinoma. For instance, miR-125b was reported upregulated in hematological malignance and prostate cancer, where it seemed to act as oncogene [6, 7]. In contrast, miR-125b was described downregulated in ovarian, bladder and breast carcinomas, assuming as a tumour suppressor [8–10]. Our recent study based on the analysis of Chip and qRT-PCR data revealed that miR-125b was upregulated in colorectal cancer with lymph node metastasis [11], which is consistent with Baffa's report [12]. However, there is no research that discloses the functional mechanism of miR-125b in the development of CRC.

Granulocyte colony-stimulating factor (G-CSF), a member of hematopoietic cytokine, acts on G-CSFR to promote the proliferation and differentiation of bone marrow cells as well as mediating their migration from bone marrow to the blood [13]. G-CSF and G-CSFR are aberrantly expressed in diverse human tumors including gastric, colon, glioma, esophageal and bladder cancer [14–17]. The combination of G-CSF with G-CSFR could active many downstream signaling pathways and further promote tumor angiogenesis, proliferation, and metastasis, although the detailed mechanism remains unclear [14–18]. A recent study in murine 32D cells showed that many miRNAs, including miR-125b, were up-regulated after G-CSF treatment. Overexpression of miR-125b enabled G-CSF–dependent proliferation [19]. This study suggested a correlation between G-CSF and miR-125b, which attracted our attention to a further investigation.

Myeloid cell leukemia 1(MCL1) belongs to Bcl2 family, which contributes to regulate both cell viability and proliferation through interaction with signal transduction networks [20]. Recently, a few studies demonstrated that MCL1 was displayed differently in carcinoma *in situ* and metastatic sites [21, 22], suggesting that MCL1 may participate in tumor metastasis. However, the relationship between MCL1 and CRC metastasis has not been revealed.

In this study, we found that miR-125b was significantly downregulated in CRC tissues from patients without lymph node metastasis while upregulated in those with lymph node metastasis. G-CSF induced miR-125b suppressed CRC cell proliferation but promoted their migration and invasion. Furthermore, we identified that miR-125b plays a pro-metastatic effect through inhibiting the expression of its target gene MCL1. G-CSF/miR-125b/MCL1 signal pathway may provide promising therapeutic targets for CRC patients.

## RESULTS

### The expression of miR-125b was associated with CRC development

**Figure 1:**
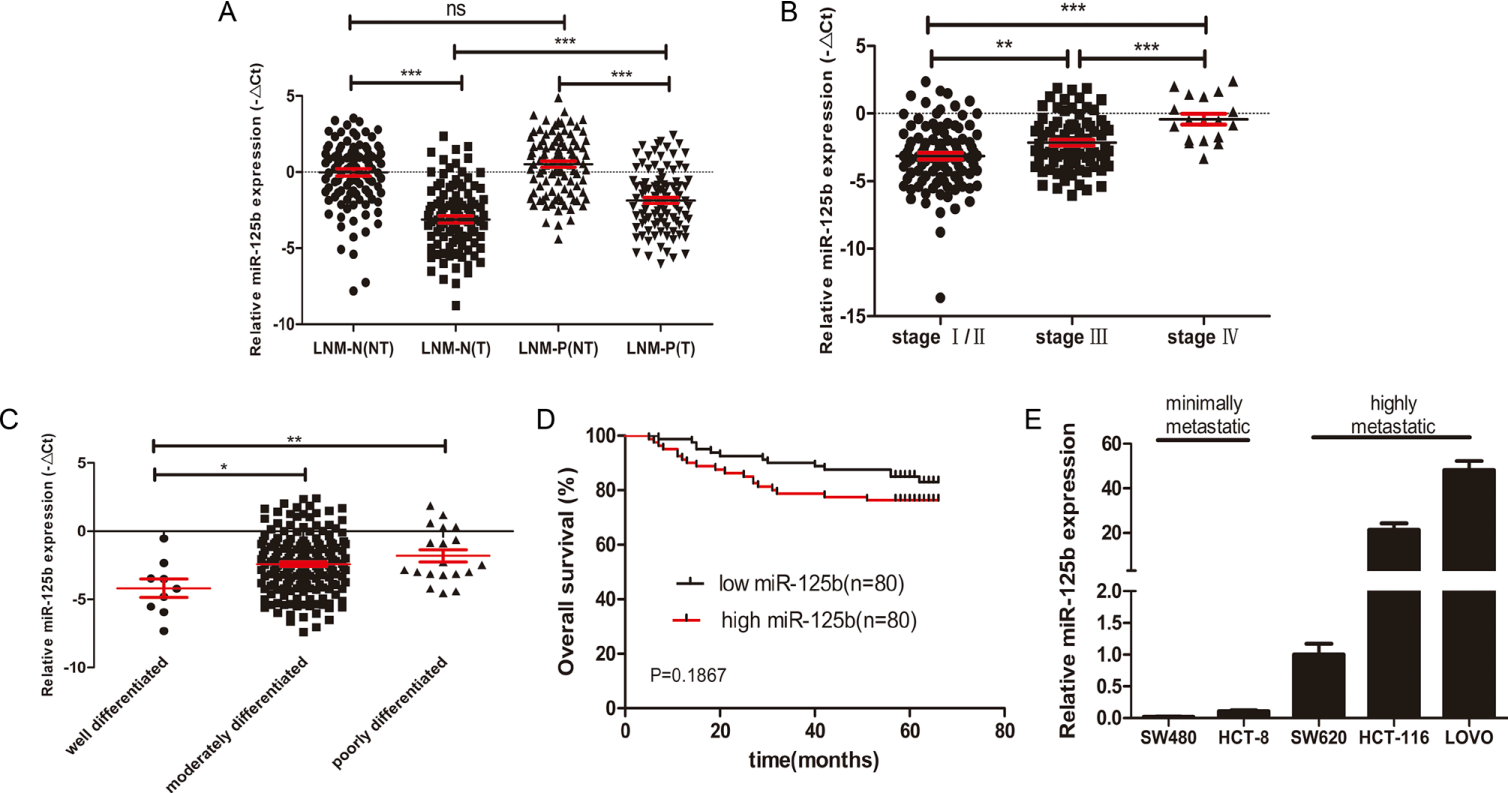
High expression level of miR125b was related to metastasis, advanced stages, poor differentiation and poor survival of CRC (**A**) qRT-PCR analysis of the relative expression of miR-125b in 202 cases of CRC patients with (LNM-P, *n* = 99) or without (LNM-N, *n* = 103) lymph node metastasis. T: tumor sample, NT: matched adjacent normal tissue. (**B**) The expression of miR-125b was compared according to the clinical stages in CRC tissues. (**C**) The expression of miR-125b was compared according to the degree of differentiation in CRC tissues. (**D**) Tissue samples of 160 CRC patients were classified into high expression group (*n* = 80) and low expression group (*n* = 80) by the median of miR-125b expression in tumor tissues. Kaplan-Meier overall survival curves of the two group of patients were shown. (**E**) The expression of miR-125b in CRC cell lines with different metastatic potential. *P < 0.05 **P < 0.01 ***P < 0.001.

To elucidate the miR-125b expression profile in CRC, we performed qRT-PCR in 202 individual pairs of CRC tissues and their matched adjacent normal tissues. The results showed that miR-125b expression was down-regulated in tumor tissues compared with that in adjacent normal tissues (−2.493 ± 0.162 vs. 0.249 ± 0.159) (Figure 1A). However, the expression level of miR-125b was remarkable upregulated in CRC tissues with lymph node metastasis than in those without lymph node metastasis (−1.853 ± 0.201 vs. −3.108 ± 0.237) (Figure 1A). In addition, the results showed that the expression of miR-125b was significantly increased in patients in advanced stages or with poor differentiation (Figure 1B, 1C). The correlation between miR-125b expression and clinical features indicated that miR-125b involved in the progression of CRC (See Table 1). The five-year survival of 160 out of 202 patients was analyzed. The Kaplan-Meier method and Log-rank test analysis found that the high expression of miR-125b was associated with the poor overall survival of CRC patients although the trend is not significant (*p* = 0.1867, Figure 1D). Unfortunately, multivariate Cox regression analysis indicated that miR-125b was not an independent prognostic factor for CRC patients (Supplementary Table 1), maybe because the five-years follow-up period was not long enough. We further determined the expression level of miR-125b in human CRC cell lines with high (SW620, HCT116, and LoVo) and low (HCT-8 and SW480) metastatic potential. The expression of miR-125b was relatively high in the former and low in the later respectively (Figure 1E). These further confirmed that miR-125b might contribute to CRC metastasis and progression.

**Table 1:** Clinicopathological associations of miR-125b expression in CRC

Clinical characteristics	case numbers (%)	miR-125b expression		*P*-value
		low (*n* = 101)	high (*n* = 101)	
**Age(year)**				1
< 60	98 (48.5%)	49	49	
≥ 60	104 (51.5%)	52	52	
**Gender**				0.06
Male	117 (57.9%)	52	65	
Female	85 (42.1%)	49	36	
Location				0.73
Colon	92 (45.5%)	42	50	
rectum	110 (54.5%)	59	51	
**Tumor size**				0.09
< 5	104 (51.5%)	58	46	
≥ 5	98 (48.5%)	43	55	
**Differentiation**				0.14
Well	10 (5%)	8	2	
Moderate	172 (85.1%)	84	88	
Poor	20 (9.9%)	9	11	
Lymph node metastasis				< 0.01**
Negative	103 (51%)	62	41	
Positive	99 (49%)	39	60	
**TNM stage**				< 0.001***
I	35 (17.3%)	23	12	
II	66 (32.7%)	39	27	
III	83 (41.9%)	38	45	
IV	18 (8.9%)	1	17	

*P-value < 0.05 **P-value < 0.01 ***P-value < 0.001.

low: ΔCt < −2.53 high: ΔCt > −2.53.

### MiR-125b inhibits proliferation, promotes apoptosis and blocks cell cycle of CRC cell

**Figure 2:**
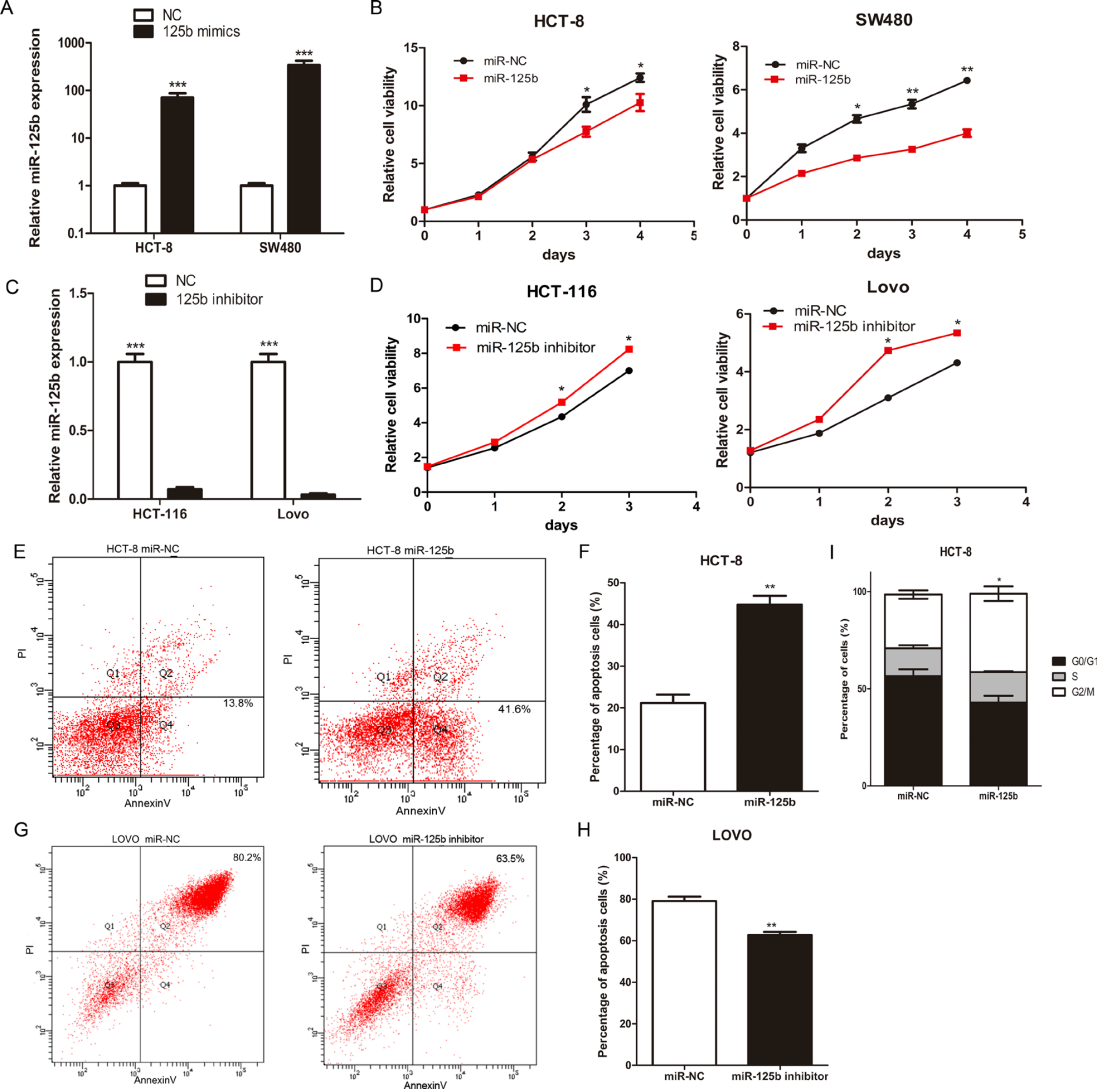
MiR-125b inhibited proliferation, promoted cell apoptosis and blocked cell cycle of CRC cells (**A**) The expression level of miR-125b after transfected with miR-125b mimics for 48 hours in HCT-8 and SW480 cell lines. (**B**) Growth curves of HCT-8 and SW480 cells transfected with miR-125b mimics or negative control. (**C**) The expression level of miR-125b after transfected with miR-125b inhibitor for 48 hours in HCT-116 and LOVO cell lines. (**D**) Growth curves of HCT-116 and LOVO cells transfected with miR-125b inhibitor or negative control. (**E**–**H**)The percentage of early (Q4) and late (Q2) apoptotic cells was shown. LOVO cells were under the condition of serum free medium 36 hours. (**I**)The percentage of cells in the cell cycle of HCT-8 cells transfected with miR-125b mimics or negative control. *P < 0.05 **P < 0.01 ***P < 0.001.

To observe the biological function of miR-125b on CRC cell, we selected HCT-8 and SW480 cells, both with relatively lower endogenous miR-125b, for the transfection of miR-125b mimics (Figure 2A). Then we selected HCT-116 and LOVO, both with relatively higher endogenous expression of miR-125b, for the transfection of miR-125b inhibitor (Figure 2C). MTT assays revealed that overexpression of miR-125b significantly suppressed the growth of CRC cells *in vitro* whereas inhibition of miR-125b promoted the growth of CRC cells (Figure 2B, 2D). Then we analyzed the effect of miR-125b on cell apoptosis and cycle using a flow cytometry assay. The result showed that overexpression of miR-125b significantly promoted HCT-8 apoptosis (Figure 2E, 2F) and blocked its cycle progression into G2/M stage in parallel (Figure 2I) while down-regulation of miR-125b inhibited LOVO apoptosis after cultured with serum free medium 36 hours (Figure 2G, 2H). These results suggested that miR-125b inhibited CRC cell proliferation through promoting the apoptosis and blockage of cycle progression.

### MiR-125b promoted CRC invasion and migration *in vitro* and *in vivo*

**Figure 3:**
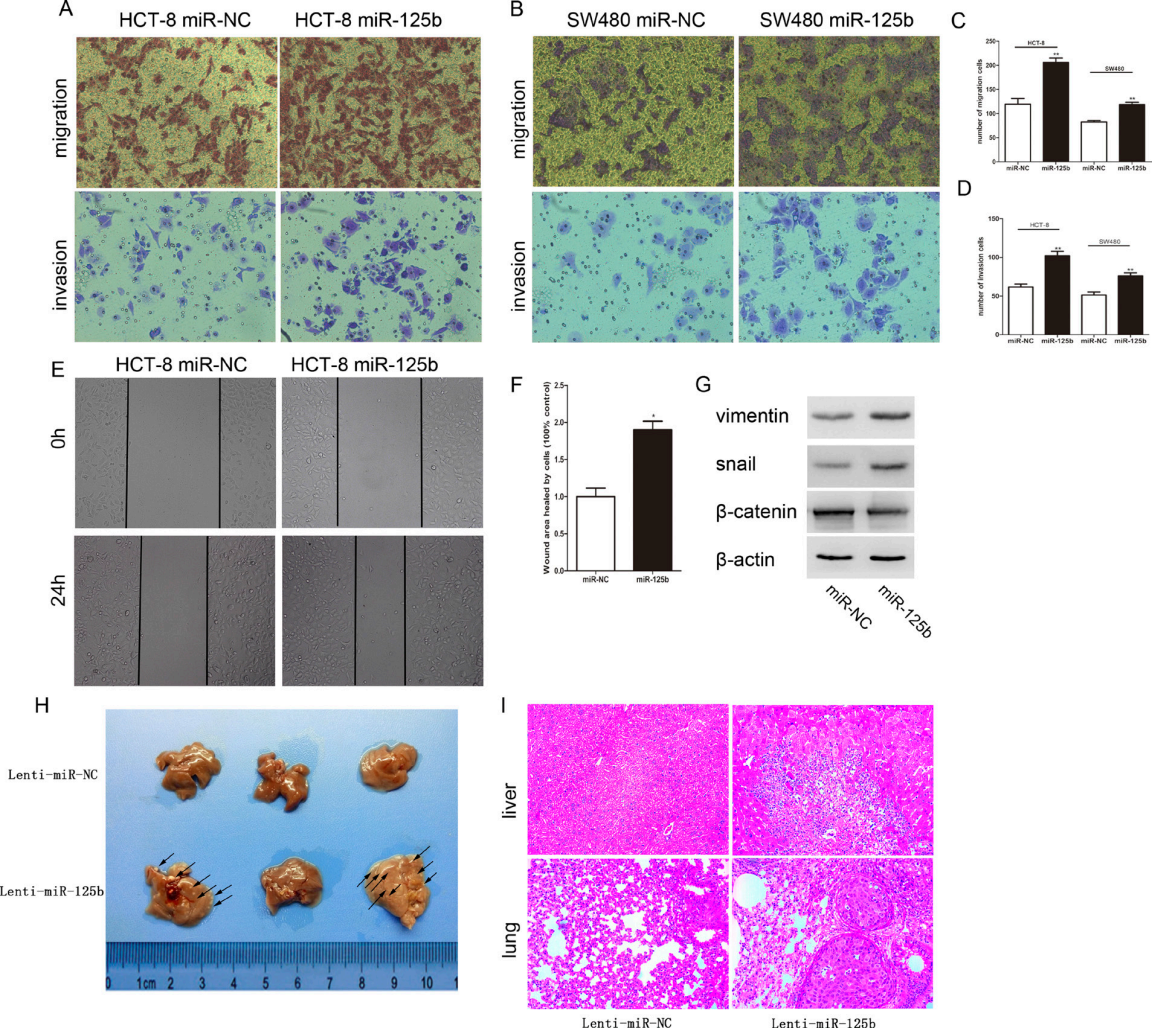
MiR-125b promoted the migration and invasion of CRC cells *in vitro* and *in vivo* (**A**, **B**) The migration and invasion of HCT-8 and SW480 cells were analyzed by transwell assay. Transwell migration assays revealed that ectopic overexpression of miR-125b promoted the migration of HCT-8 and SW480 cells. Transwell invasion assay with Matrigel-coated membranes revealed that overexpression of miR-125b promoted the invasion of HCT-8 and SW480 cells. (**C**, **D**) The cell numbers of migration and invasion were compared between groups of miR-125 overexpression (miR-125b) and control (miR-NC). (**E**) The “wound” was created when the cells became confluent monolayer. The images were captured at the beginning and 24 hours thereafter. (**F**) Comparison of the cells to quantify the confluent rate of the two group cells. (**G**) The expression of EMT marker in HCT-8 cells with or without ectopic overexpression of miR-125b. (**H**) Impact of miR-125b over-expression CRC cells on mice liver metastasis. (**I**) The images of normal liver or lung tissue and metastatic colony tissue stained by HE section were presented (magnification × 200). *P < 0.05 **P < 0.01.

To disclose the relationship between the expression level of miR-125b and lymph nodes metastasis, we ectopically expressed miR-125b in HCT-8 and SW480 to evaluate the effect of miR-125b on the migration and invasion ability of CRC cells. Transwell migration array and matrigel invasion array indicated that ectopic miR-125b could significantly promote migration and invasion of CRC cells respectively (Figure 3A–3D). Also, the migration capacities were evaluated via wound healing assay, which demonstrated that the migration speed of miR-125b-overexpressed HCT-8 cells into the wound was much faster than that of the control group (Figure 3E, 3F). Furthermore, the pro-metastasis role of miR-125b was studied in animal models by tail intravenous injection of colorectal cancer cells. The results showed the mice injected with miR-125b over-expression CRC cells appeared tumor metastasis in liver and lung comparing to the control group. Epithelial-mesenchymal transition (EMT) was known to participate with the tumor metastasis. Thus we testified if miR-125b could promote EMT process. As we expected, Western blot assay showed that the mesenchyme cell marker Vimentin and Snail was significantly upregulated, while the epithelial cell marker β-catenin was significantly downregulated in miR-125b overexpressed HCT-8 cell line (Figure 3G). Taken together, miR-125b could promote EMT process of CRC cells.

### MCL-1 is a direct target of miR-125b

**Figure 4:**
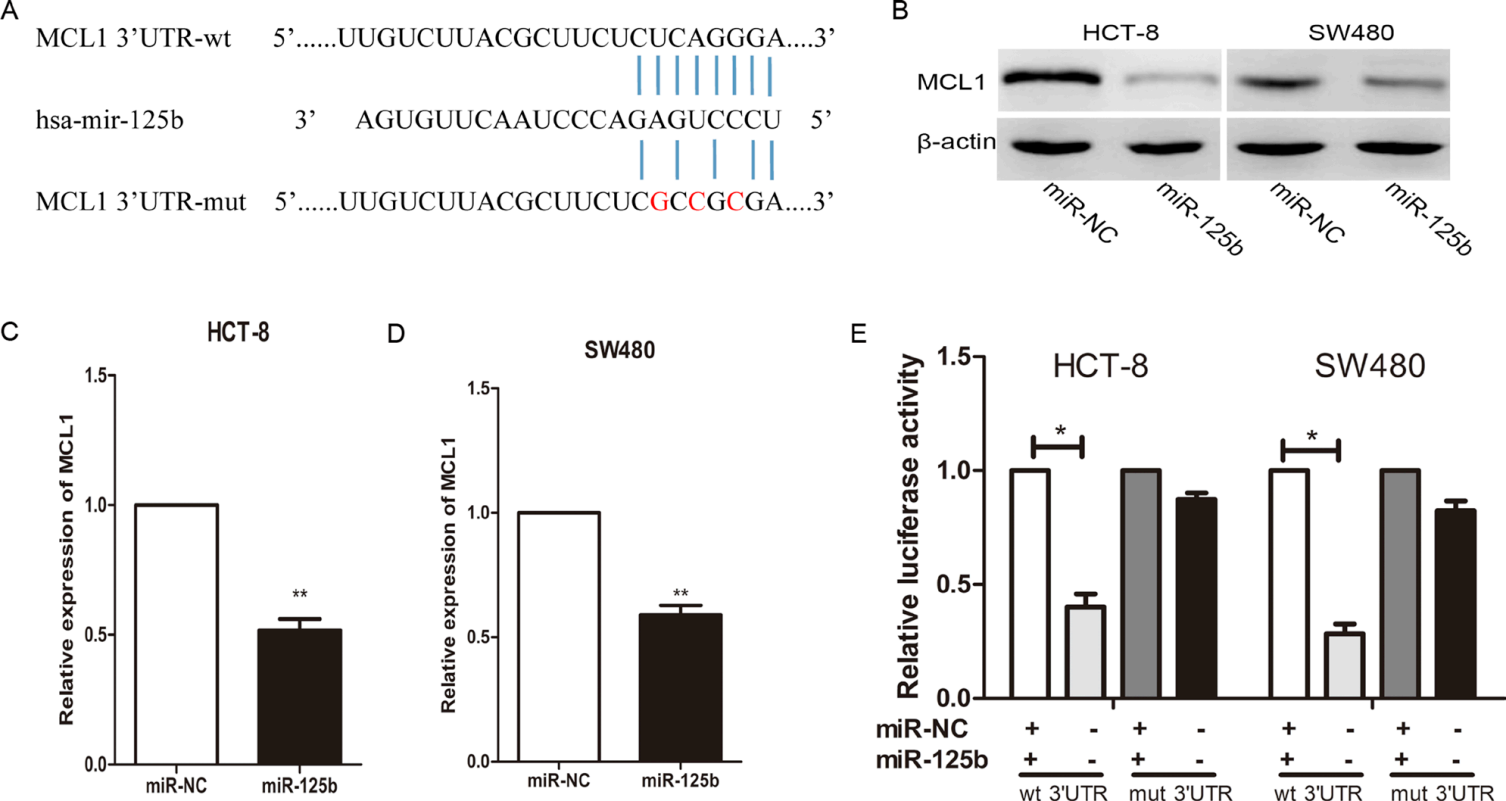
MiR-125b inhibits MCL1 expression by directly binding to its 3′-UTR in CRC cell lines (**A**) The predicted sites of miR-125b binding to the 3′-UTR region of MCL1 were detected using bioinformatics prediction tools. The mutated site in the 3′-UTR region of MCL1 is shown. (**B**, **C**, **D**) MCL1 in both the protein and mRNA level was decreased after the transfection with miR-125b mimic in HCT-8 and SW480 cell lines as demonstrated by Western blot and qRT-PCR respectively. (**E**) The effect of miR-125b on the luciferase activity induced by pMIR-MCL1-wt or pMIR-MCL1-mut reporter plasmids in HCT-8 and SW480 cells was measured via luciferase reporter gene assays. *P < 0.05 **P < 0.01.

To explore the molecular mechanisms responsible for the pro-metastasis effect of miR-125b, we searched for candidate target genes using bioinformatic software such as miRanda, miRDB, and TargetScan. Among hundreds of potential candidates, we focused on the genes which involved in tumor metastasis. MCL1 attracted our attention since its 3′-UTR contains putative target sequences for miR-125b (Figure 4A). In addition, MCL1 was involved in proliferation, apoptosis and metastasis of several cancer cell lines [20–22]. Although MCL1 has been reported participating in the apoptosis and drug resistance of CRC cells induced by miR-125b [23, 24], there is no report about its contribution to miR-125b induced metastasis. After exogenetic expressing miR-125b in HCT-8 and SW480 cell, the expression of MCL1 in both protein and mRNA level was decreased (Figure 4B–4D). To confirm whether MCL-1 was the direct target of miR-125b, we performed a dual-luciferase activity assay. We constructed the wild type or mutated 3′ UTR sequence of MCL1 to the firefly luciferase vector (Figure 4A). Then the vector was co-transfected with miR-125b mimics, and the luciferase expression was measured. As shown in Figure 4E, miR-125b was able to markedly inhibit the relative luciferase activity with the wild-type MCL1 3′-UTR, but did not change the activity of the mutant MCL1 3′-UTR constructs. The result suggested that miR-125b inhibited MCL1 expression via the binding site in MCL1 3′UTR. Taken together, these data demonstrated that miR-125b could attenuate the expression of MCL1 by directly targeting its 3′-UTR.

### MCL1 reverses the effect of miR-125b on the migration and invasion of CRC cells

**Figure 5:**
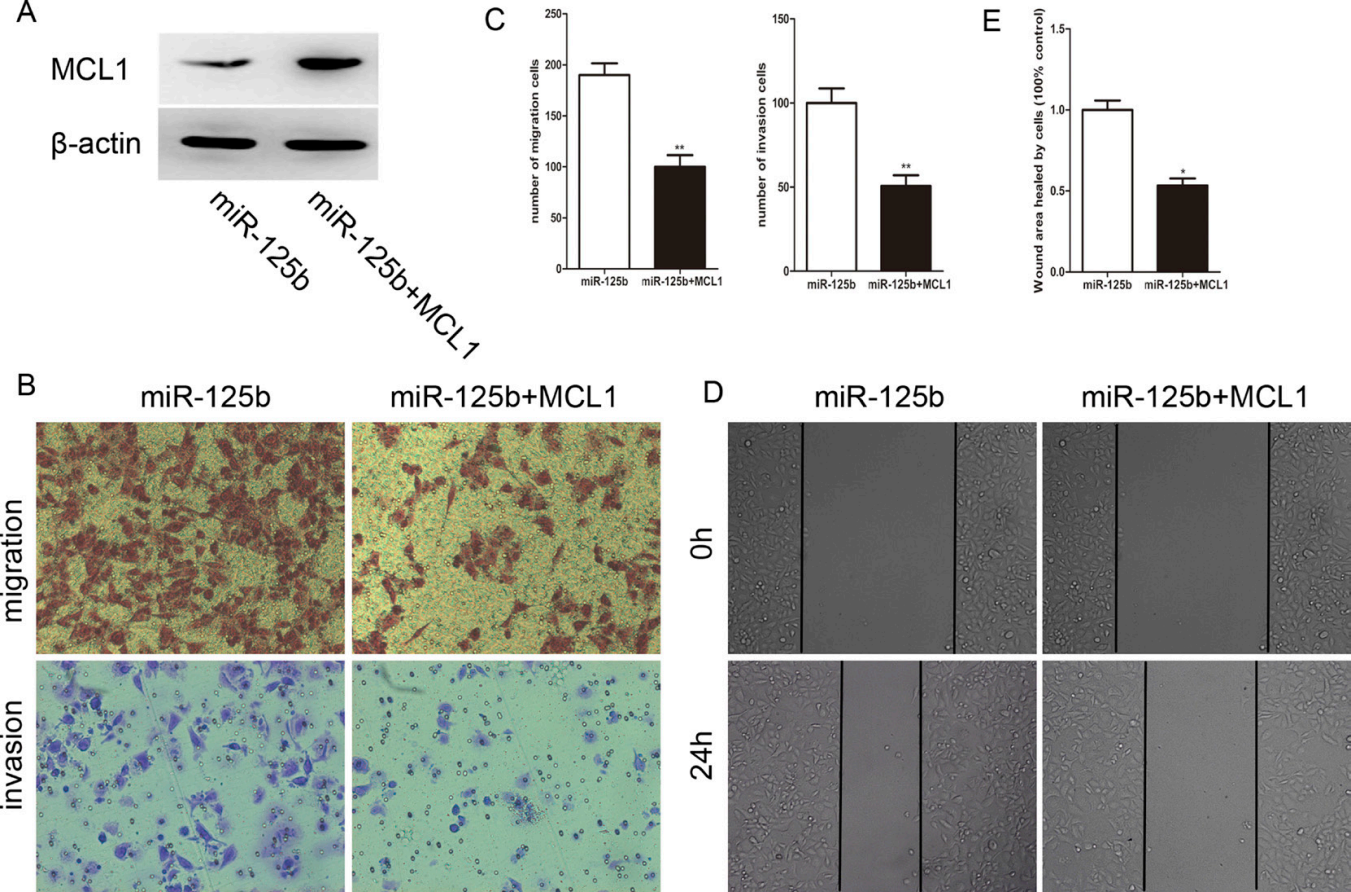
Reexpression of MCL1 reverses the effect of migration and invasion induced by miR-125b in HCT-8 (**A**)The expression level of MCL1 in HCT-8 cells after co-transfecting with pcDNA-MCL1 and miR-125b. (**B**) The miR-125b induced migration and invasion of CRC cells were restored via MCL1 reexpression. (**C**) The cell numbers of migration and invasion between two groups. (**D**) MCL1 attenuated wound healing ability of HCE-8 cells overexpressed with miR-125b. (**E**) The ratio of healing compared between two groups at 24 h after creating a wound. *P < 0.05 **P < 0.01.

To further investigate whether the pro-metastasis role of miR-125b was directedly mediated by MCL1, a gain-of-function study was performed. We constructed a vector containing the MCL1 open reading frame (ORF) without its 3′UTR sequence. After the transfection of MCL1 ORF into HCT-8 cells, the protein level of MCL1 was detected (Figure 5A). As shown in Figure 5B, 5C, overexpression of MCL1 significantly abolished the effects of miR-125b on cell migration and invasion. Additionally, wound healing assay derived the same conclusion (Figure 5D, 5E). Collectively, these data strongly indicate that MCL1 is the direct pro-metastasis mediator of miR-125b.

### Protein level of MCL1 was down-regulated in CRC tissue with lymph node metastasis

**Figure 6:**
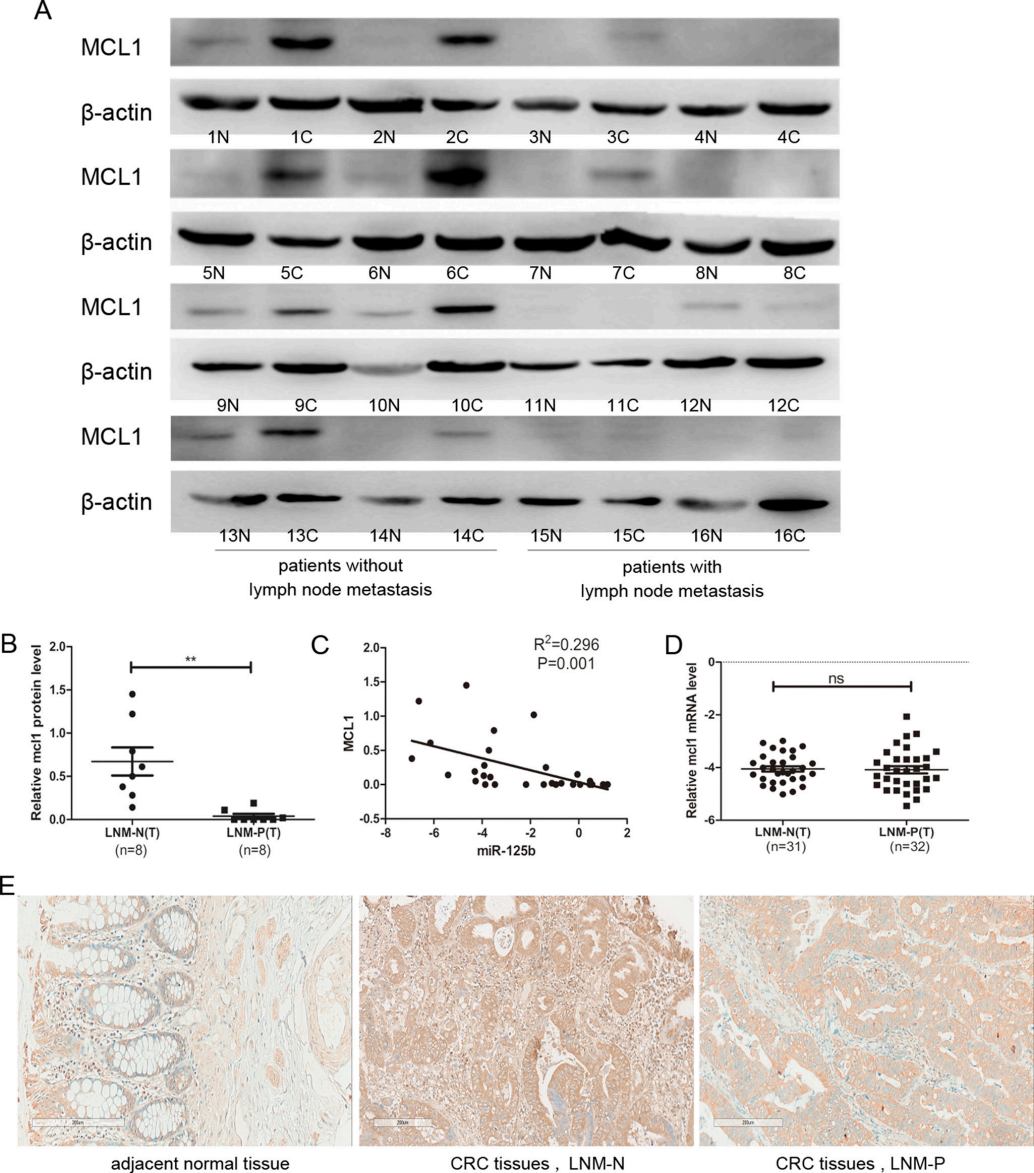
The protein expression level of MCL1 was downregulated in CRC tissue with lymph node metastasis compared to those without lymph node metastasis (**A**) Western blot analyzed the relative protein expression of MCL1 in 16 cases CRC patients, including tumor sample (c) and matched adjacent normal tissues (n) from the same patient. (**B**) Statistical analysis for the protein expression of MCL1 in CRC tissues with the lymph node metastasis negative (LNM-N) or positive (LNM-P). (**C**) The correlation between miR-125b and protein expressions in 16 colorectal cancer patients. (**D**) qRT-PCR analysis of the mRNA expression of MCL1 in CRC tissues with the lymph node metastasis negative (LNM-N) or positive (LNM-P). (**E**) Immunohistochemiatry analyzed the relative protein expression of MCL1 in CRC patients, including tumor sample (c) and matched adjacent normal tissues (n) from the same patient. *P < 0.05 **P < 0.01.

To further investigate the relationship between MCL1 and CRC metastasis, we examined protein expression level of MCL1 in 16 CRC patients using Western Blot (8 cases with lymph node metastasis and 8 cases without lymph node metastasis). As shown in Figure 6A and 6B, the expression of MCL1 was significantly down-regulated in CRC tissues with lymph node metastasis compared to those without lymph node metastasis, which is negatively related to the miR-125b expression level (Figure 6C). Interestingly, the mRNA level of MCL1 had no difference in tissues no matter with lymph node metastasis or not (Figure 6D), which further proved that miR-125b inhibited MCL1 translational process through binding its mRNA 3′UTR rather than inhibiting in mRNA level. The expression level of MCL1 was confirmed by immunohistochemiatry experiment in CRC tissues (Figure 6E) and arrived at the same conclusion.

### G-CSF promoted miR-125b expression in CRC cells

Reviewing the data of miR-125b expression in CRC tissues, it is evident that miR-125b went through a progression from downregulated to upregulated. This drove our attention to the research of modulators that stimulated miR-125b expression.

**Figure 7:**
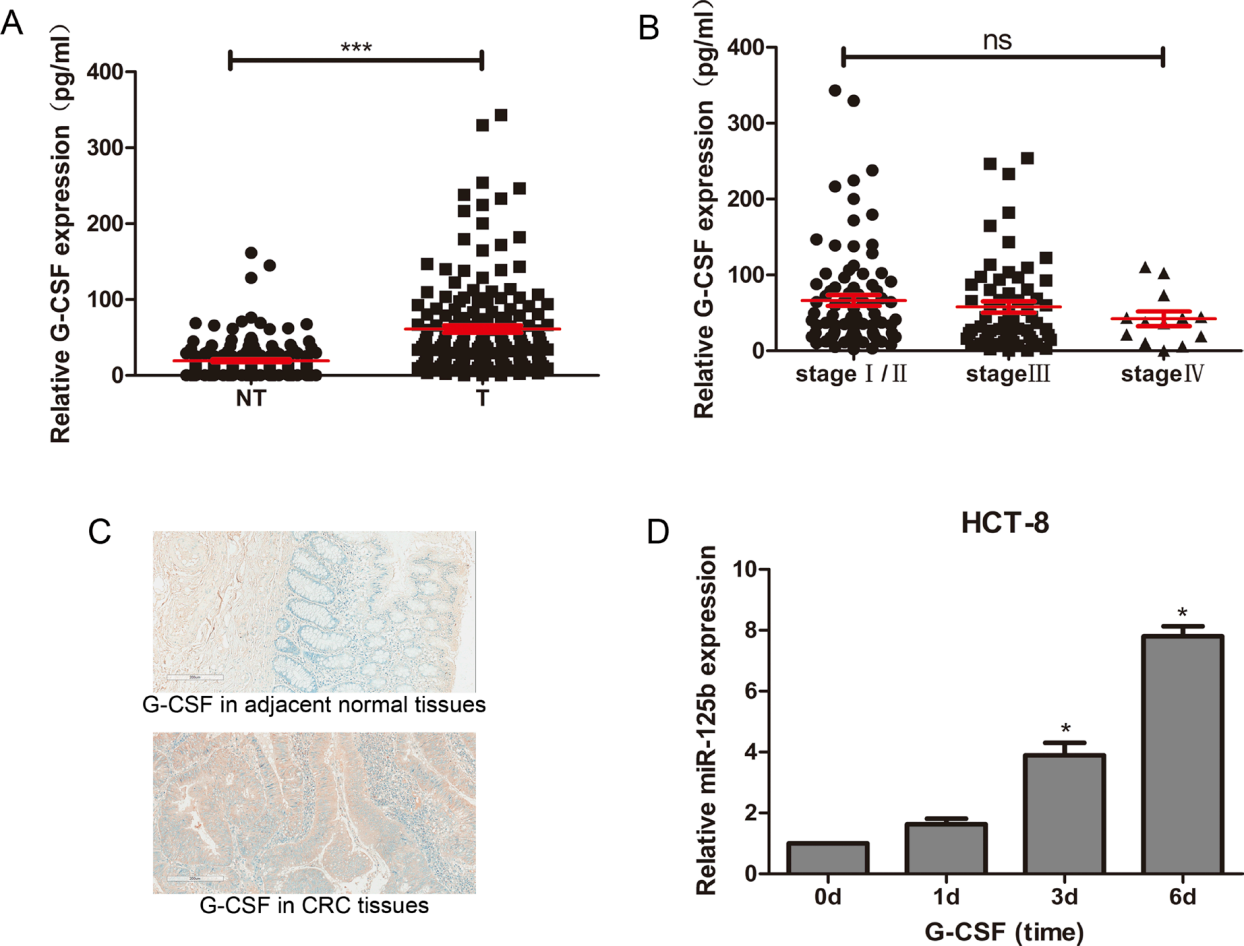
G-CSF promoted expression of miR-125b in HCT-8 and upregulated in CRC tissues (**A**) ELISA assay of G-CSF expression in CRC tissues (T, *n* = 163) and adjacent normal tissues (NT, *n* = 163). (**B**) ELISA assay of G-CSF expression in CRC tissues at different stages (stage I/II *n* = 88, stage III *n* = 62, stage IV *n* = 13). (**C**) Immunohistochemiatry assay of G-CSF expression in CRC tissues comparing to adjacent non-tumor tissues. (**D**) qRT-PCR analysis of miR-125b expression in HCT-8 stimulated with 50 ng/ml G-CSF on day 0, day1, day 3 and day 6. *P < 0.05 **P < 0.01 ***P < 0.001.

Considering the promotive effect of G-CSF on the expression of miR-125b in Surdziel's study [19], we evaluated the expression of G-CSF in CRC tissues. We compared protein level of G-CSF in 163 individual CRC tissues to their matched adjacent normal tissues. The result showed a significant upregulation in CRC tissues (Figure 7A). The expression level of G-CSF was confirmed by immunohistochemiatry experiment, which showed the consistent results (Figure 7C). Interestingly, G-CSF displayed a similar expression level between different stages of CRC tissues (Figure 7B), indicating that the continued stimulation with G-CSF leads to upregulation of miR-125b in CRC tissues in advanced stages.

An *in vitro* experiment was designed to proof this hypothesis. HCT-8 Cells was cultured in the medium with 50ng/ml G-CSF. The expression of miR-125b in HCT-8 cells was detected on 0, 1, 3 and 6 days. As we observed, the expression level of miR-125b increased gradually under G-CSF stimulation (Figure 7D).

## DISCUSSION

Dysregulation of miRNAs has been reported to be closely associated with tumor initiation and progression in diverse cancers including colorectal cancer (CRC). Some miRNAs are responsible for CRC development, like miR-92a, miR-96, miR-135a/b and miR-224 [25–28], whereas the others (miR-7, miR-26b and miR-101) are suppressive factors [29–31].

Our previous study identified several differentially expressed microRNAs in CRC tissues with or without lymph node metastasis [11]. MiR-125b was one among them, which was reported up-regulated in hematological malignancy and prostate cancer [6, 7] while down-regulated in ovarian, bladder and breast cancer [8–10]. These indicated that miR-125b might play either an oncogenic or tumor-suppressive role in different types of cancer. In CRC, miR-125b was reported down-regulated in patients without lymph node metastasis while up-regulated in those with lymph node metastasis [32–35]. However, all of these results were based on data in RNA level from miRNA microarray or qRT-PCR, and the difference was not significant enough because of the deficiency of samples. Therefore, the exact mechanism of miR-125b on CRC initiation and progression remain unknown.

In the present study, we used a total of 202 paired samples from CRC patients (103 with lymph node metastasis and 99 without lymph node metastasis) to further investigate the role of miR-125b in cancer development. We demonstrated that the expression of miR-125b was remarkably down-regulated or up-regulated in CRC tissues without or with lymph node metastasis respectively. CRC cell lines with high expression level of miR-125b also presented high metastasis potentiality. High expression of miR-125b was associated with the disease progression and poor overall survival of CRC patients, which was consistent with the previous study [36]. However, multivariate analyses indicated that miR-125b was not an independent prognostic factor for CRC patients in our study. Whether miR-125b can be served as an independent prognostic biomarker for CRC patients still needed to be further validated with a longer follow-up period. Furthermore, the functional study showed that miR-125b inhibited cell growth, triggered cell cycle arrest, induced cell apoptosis and promoted cell migration and invasion. These results suggested that miR-125b acted as a tumor suppressor in CRC initiation, but an oncogene was contributing to the progression and metastasis of CRC later on. Targeting miR-125b may provide a potential treatment to cease CRC development. The mechanism behind this is that miR125b silenced MCL-1. MCL-1 is one of the essential anti-apoptotic factors in the physiological process of normal cells. Recent reports had shown that MCL-1 was over-expressed in several tumors, including chronic myeloid leukemia, lung cancer, and hepatocellular carcinoma [37–40], suggesting that MCL-1 may participate in the tumor initiation and can be a potential therapeutic target in the treatment of several human malignancies. MCL-1 was shown to be negatively regulated by microRNAs that resulting in the attenuation of its anti-apoptosis roles. For instance, over-expressing miR-29b reduced MCL-1 cellular protein levels and increased TRAIL (tumor necrosis factor-related apoptosis-inducing ligand)-induced apoptosis accordingly [41]. In addition, it has been reported that miR-125b was able to promote apoptosis in various cancer cell lines by direct or indirect targeting of MCL1 [42]. There are also reports about MCL1 and tumor metastasis, although the mechanism remains unclear [21, 22].

Here we revealed that the protein expression level of MCL1 was down-regulated in CRC tissues with lymph node metastasis comparing to those without lymph node metastasis. We found that MCL1 mRNA was directly targeted by miR-125b. Both of the mRNA and protein expression levels of MCL1 were reduced by miR-125b in CRC cells. Furthermore, restoring expression of MCL1 attenuated invasion and migration-promoting effect induced by miR-125b, indicating that MCL1 functions as a mediator of miR-125b on promoting CRC metastasis. However, the mRNA expression level of MCL1 displayed no difference in CRC tissue with or without lymph node metastasis, suggesting that miR-125b only influenced the protein expression level of MCL1. Through which signal pathway that MCL1 participated in tumor metastasis and whether other promising targets of miR-125b existed in colorectal cancer need a further investigation.

G-CSF was reported to promote carcinoma cells proliferation and migration, but the mechanism was still under investigation [14, 15]. The previous study showed that G-CSF was able to up-regulate miR-125b expression in murine 32D cells [19]. Thus we hypothesize that miR-125b could be upregulated by G-CSF in CRC cells as well; since G-CSF was significantly up-regulated in CRC tissues comparing to adjacent normal tissues. As we expected, the expression level of miR-125b was elevated in CRC cells cultured in a medium conditioned with 50 ng/ml G-CSF. This could explain the phenomenon of miR-125b expression experienced from down-regulation to up-regulation along with CRC development. Therefore we came to a conclusion that the expression of miR-125b could be induced by G-CSF in CRC tissues, which resulted in increased mobility in the advanced stage.

In summary, we found that miR-125b was down-regulated in CRC initiation while up-regulated in CRC progression stimulated by G-CSF. High expression of miR-125b inhibited proliferation but promoted metastasis by direct targeting to MCL1. Therefore, miR-125b may serve as a biomarker for CRC prognosis. The further study of G-CSF/miR-125b/MCL1 pathway might provide potential therapeutic targets for the control of metastasis of CRC.

## MATERIALS AND METHODS

### Clinical samples

All the clinical specimens used in this study were obtained from 202 patients in Cancer Hospital Chinese Academy of Medical Sciences (Beijing, China). All of the patients underwent surgical resection at 2011, and none of the patients received chemotherapy or radiotherapy before surgery. Resected tissues were histologically examined by H&E staining. Fresh CRC tissues and matched cancer-adjacent normal tissues were immediately collected after surgical removal and snap-frozen in liquid nitrogen for further use. The study obtained the written informed consent from all patients and was approved by the Ethics Committee of Cancer Hospital Chinese Academy of Medical Sciences. The clinical characteristics of these patients are shown in Table 1.

### Cell culture and transfection

The human CRC cell line HCT-8, SW480, SW620, HCT-116, LOVO was purchased from Institute of Basic Medical Sciences Chinese Academy of Medical Sciences’ cell culture center (Beijing, China). These cells were cultured in a humidified 5% CO2 atmosphere at 37°C and incubated in RPMI 1640 or L15 containing 10% fetal bovine serum (Gibco, CA). A miR-125b mimic and a negative control oligonucleotides were purchased from Genechem biology company (Shanghai, China). Transfection of oligonucleotides was performed using Lipofectamine 2000 (Invitrogen) according to the manufacturer′s protocols.

### RNA extraction and qRT-PCR

Total RNA from cultured cells or tissues was extracted using the Qiagen miRNeasy Mini Kit according to the manufacturer›s instructions. Reverse transcription reactions were performed using MiScript Reverse Transcription Kit (Qiagen, Germany.). Mature miRNA was detected using MiScript SYBR Green PCR Kit in combination with specific primers (synthesis in Ribobio). LightCycler 480 (Roche, Basel, Switzerland) was used to detect mature miRNAs and mRNAs. U6 and β-actin were used as miRNA and mRNA internal control respectively. The relative expression of miRNA compared with U6 was calculated using the −ΔCt or 2^−ΔΔCt^ method. All qRT-PCR reactions were performed in triplicate.

### Proliferation assay

24 h later after transfection, cells were seeded on a 96-well plate at a density of 2 × 10^3^cells/well, grown at 37°C, and then cell viability was tested every 24 hours using the cell counting MTT. Briefly, 50 μl of MTT (5 mg/ml in PBS) was added to each well, and the cells were incubated for a further 4 h. After removal of the medium, 100 μl of DMSO was added to each well and vibrate it slowly for 10 minutes. The absorbance was finally determined at 450 nm using a microplate reader.

### Cell apoptosis assays and cell cycle analysis

Apoptosis was measured using the FITC Annexin V apoptosis detection kit I (BD Biosciences). Forty-eight hours after miR-125b transfection, cells were incubated with Annexin V-fluorescein isothiocyanate (FITC) and PI, and the percentage of apoptotic cells was analyzed by flow cytometry. For cell cycle analysis, cells were washed and fixed with ice-cold 75% ethanol at −20°C for 2 h, then stained with PI at the concentration of 50 mg/mL. DNA content was analyzed by Flow cytometry analysis.

### Migration, invasion and wound healing assay

Migration assay and invasion assay were performed by transwell. Briefly, 5 × 10^4^cells were plated in the serum-free medium in the upper chamber with the non-coated membrane (24-well insert; pore size, 8 mm; BD Biosciences) for migration assay and with Matrigel-coated membrane (24-well insert; pore size, 8 mm; BD Biosciences) for invasion assay. The lower chamber was filled with medium containing 10% fetal bovine serum. The cells were incubated for 24 h at 37°C. Then the cells on the upper surface were removed by cotton swab and the cells on the lower surface were stained with crystal violet and observed under a microscope. For wound healing assay, a wound was made after the cells grow confluence. After wounding, cells were washed at least twice and added new culture medium without fetal bovine serum. The degree of healing was measured 24h later using an inverted microscope.

### ELISA

The protein expression level of G-CSF in CRC tissues was detected by Elisa kit (R&D). Briefly, coat a 96-well microplate with 100 μl per well of the diluted Capture Antibody. Seal the plate and incubate overnight at room temperature. Block plates by adding 300 μl of reagent diluent to each well. Incubate at room temperature for a minimum of 1 hour. Add 100 μl of sample or standards in reagent diluent and incubate 2 hours at room temperature. Add 100 μl of the detection antibody, diluted in reagent diluent with NGS to each well and incubate 2 hours at room temperature. Add 100 μl of working dilution of streptavidin-HRP to each well and incubate for 20 minutes at temperature. Add 100 μl of substrate solution to each well and incubate for 20 minutes at room temperature. Add 50 μl of stop solution to each well. Determine the optical density of each well immediately, using a microplate reader set to 450 nm.

### Western blot

Cells were washed twice with PBS. Proteins were extracted from the cells using the lysis buffer and were quantified with BSA as a standard, then separated by 10% SDS-PAGE. After transferred to a nitrocellulose membrane, the membrane was incubated with primary antibody: anti-MCL1 [Y37, Abcam], anti-vimentin [EPR3776, Abcam], anti-snail [C15D3,CST], anti-β-catenin [D10A8,CST] and anti-β-actin [A5441,Sigma]. And then the membrane was incubated with secondary antibody. After washed three times with TBST, the intensities of the target proteins were quantified using an image analysis system.

### Luciferase reporter assays

To confirm miR-125b direct targets the 3′UTR of MCL1, the wild-type or mutant 3′UTR sequence of MCL1 with relative miR-125b specific binding sequence were transfected with pMIR-Reporter luciferase vector into HCT-8 and SW480 cells. 48 h after transfection, Luciferase assays were performed using the dual luciferase reporter assay system (Promega) and analyzed after normalization to the Firefly luciferase activity. Transfections were performed with Lipofectamine 2000 reagent (Invitrogen).

### Tumorigenesis in nude mice

Six homogeneous mice were divided into experimental and control group. The stable transfected cultured cells (2 × 10^6^/ml, 200 μl) were injected into tail vein of nude mice to evaluate the ability of liver or lung metastasis. All mice were monitored every 7 days and were sacrificed 8 weeks later. The liver or lung was exposed and grossly examined at necropsy, then carried out HE staining.

### Statistical analysis

Statistical analysis was performed using SPSS 17.0 software. Data were expressed as mean ± SD error of three repeating experiments. Student's *t*-tests were used for comparison between two groups. Survival curves were analyzed using Kaplan–Meier method with log rank test. Univariate and multivariate Cox regression analyses were applied to evaluate the hazard ratio. *P* values less than 0.05 were considered statistically significant.

## SUPPLEMENTARY MATERIALS TABLE



## Abbreviations

CRC: Colorectal cancer
G-CSF: Granulocyte colony-stimulating factor
G-CSFR: Granulocyte colony-stimulating factor receptor
MCL1: Myeloid cell leukaemia 1
EMT: Epithelial-mesenchymal transition
qRT-PCR: quantitative real-time PCR
3′UTR: 3′untranslated region
miRNA: microRNA
mRNA: messenger RNA.
