# Correction: Upregulation of microRNA-125b by G-CSF promotes metastasis in colorectal cancer

**DOI:** 10.18632/oncotarget.27377

**Published:** 2020-01-07

**Authors:** Xinghua Zhang, Xiao Ma, Huaying An, Changqing Xu, Wenjo Cao, Wei Yuan, Jie Ma

**Affiliations:** ^1^ State Key Laboratory of Molecular Oncology, National Cancer Center/National Clinical Research Center for Cancer/Cancer Hospital, Chinese Academy of Medical Sciences and Peking Union Medical College, Beijing, China; ^2^ Department of Gastroenterology, Shandong Provincial Qianfoshan Hospital, Shandong University, Jinan, China; ^3^ Department of Science, University of British Columbia, Vancouver, Canada; ^4^ Clinical Immunology Center, Chinese Academy of Medical Science, Beijing, China; ^5^ Department of Biotherapy, Beijing Hospital, National Center of Gerontology, Beijing, China

**This article has been corrected:** The 1st affiliation information has been updated. The proper affiliation is as follows:

^1^State Key Laboratory of Molecular Oncology, National Cancer Center/National Clinical Research Center for Cancer/Cancer Hospital, Chinese Academy of Medical Sciences and Peking Union Medical College, Beijing, China

Original article: Oncotarget. 2017; 8:50642–50654. 50642-50654. https://doi.org/10.18632/oncotarget.16892

