# Supplementary material for: Upregulation of microRNA-125b by G-CSF promotes metastasis in colorectal cancer
Source: Oncotarget. 2017 Apr 6;8(31):50642–54. doi: 10.18632/oncotarget.16892 (PMC5584181; doi:10.18632/oncotarget.16892)
Supplement: Supplementary file 1 [file oncotarget-08-50642-s001.pdf]

Upregulation of microRNA-125b by G-CSF promotes metastasis in colorectal cancer

Supplementary Materials

Supplementary Table 1: Summary of univariate and multivariate Cox regression analysis of overall survival duration

Factor	Univariate analysis			Multivariate analysis		
	HR	95%CI	*P*	HR	95% CI	*P*
Age	0.588	0.290~1.190	0.140			
Gender	1.162	0.568~2.377	0.682			
Location	1.756	0.873~3.530	0.114			
Tumor size	1.475	0.736~2.953	0.273			
Lymph node metastasis	0.094	0.029~0.308	< 0.001	0.590	0.171~2.036	0.404
Differentiation	4.170	1.944~9.943	< 0.001	3.422	1.520~7.702	0.003
TNM stage	4.637	2.714~7.923	< 0.001	4.750	2.462~9.162	< 0.001
miR-125b expression level	1.430	0.706~2.896	0.321			
